# Evaluation of the clinical outcomes and patient satisfaction related to the use of internal eye shields for electron external beam radiation therapy

**DOI:** 10.1002/jmrs.812

**Published:** 2024-07-15

**Authors:** Kirsty Tait, Sinead Burgess, Elizabeth A. Burmeister, Thuan Anh Le Nguyen, Bryan Burmeister

**Affiliations:** ^1^ Genesis Care Fraser Coast Hervey Bay Queensland Australia; ^2^ Wide Bay Hospital and Health Service Hervey Bay Queensland Australia; ^3^ University of Queensland Brisbane Queensland Australia

**Keywords:** electron beam, internal eye shield, Radiotherapy, regional healthcare, skin cancer around the eye

## Abstract

**Introduction:**

Cancers around the eye are often treated using orthovoltage machines or by plastic surgery, neither of which are widely available in regional Australia. External beam radiation therapy (EBRT) using electrons and an internal eye shield is an alternative, relatively underreported technique which can provide similar cosmetic and functional outcomes. This report aimed to describe the process for the use of internal eye shields at GenesisCare Fraser Coast Radiation Oncology (GCFCRO) and the associated clinical outcomes and patient perceptions of the delivery and results of this procedure.

**Methods:**

This project was conducted in two phases. Phase I was an audit of the departmental technique and short‐term clinical outcomes of 17 patients who received EBRT for skin cancer near the eyes at GCFCRO in partnership with Wide Bay Hospital and Health Service (WBHHS). Phase II was a survey of nine of those patients to elicit the patient perspective of the delivery and long‐term outcomes of the treatment.

**Results:**

Phase I revealed the departmental procedures for simulation, planning and treatment at GCFCRO are consistent with other departments published protocols. Phase II results detailed positive patient perspectives regarding cosmetic outcomes and receipt of EBRT for skin cancer near their eyes.

**Conclusion:**

EBRT with an internal eye shield is an acceptable alternative modality to surgery for squamous cell carcinomas (SCC) and basal cell carcinomas (BCC) around the eye in the definitive and adjuvant setting. This is particularly important in regional locations to facilitate patients receiving high‐quality care and outcomes locally.

## Graphical abstract

External beam radiation therapy using electrons and an internal eye shield is an alternative, relatively underreported technique which can provide similar cosmetic and functional outcomes. This report aimed to describe the process for the use of internal eye shields and the associated clinical outcomes and patient perceptions of the delivery and results of this procedure.

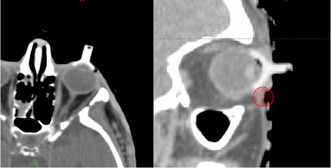

## Introduction

Cancer is Australia's leading disease burden and has a significant cost and impact on the health system.^1^ The incidence of non‐melanoma skin cancer (NMSC) in Australia, particularly in Queensland, is among the highest in the world.^2^ Treatment options for NMSC include surgery, topical therapies, radiation therapy (RT) and more recently systemic therapies.^3^

Radiation therapy provides a minimally invasive treatment option with good cosmetic outcomes and thus it is the standard of care for treatment of cancers near the mouth, nose, ears or eyes to preserve cosmesis and function.^2^, ^3^, ^4^

RT may be delivered using either a superficial voltage machine or a linear accelerator. Superficial therapy (SXT) achieves good results with minimal invasive shielding requirements; however, it is not a widely accessible treatment modality in Australia as specialised equipment is required.^5^ It is well cited in the literature that rural Australians (RA2‐5) are disproportionately affected by the burden of cancer, leading to decreased 5‐year survival rates when compared to metropolitan populations.^6^ This can be attributed to lower health literacy and socioeconomic status, higher engagement in risky behaviours such as smoking and a maldistribution of specialists and services creating practical barriers to accessing care.^1^ Cumulatively, these factors result in decreased cancer screening, lower rates of early diagnosis and decreased ability to access local care.^1^ In Queensland, most patients need to travel to metropolitan centres to access plastic and oculoplastic surgery for the treatment of skin cancers near the eye. The travel burden results in negative outcomes such as increased financial costs and psychosocial issues associated with relocating from the home environment.^7^, ^8^, ^9^

Comparatively, EBRT using electrons is more accessible, as linear accelerators are common to radiation departments in regional locations. To achieve adequate coverage of the lesion, energies within the range of 6–9 M electron voltage (MeV) are generally prescribed by the radiation oncologist (RO).^10^ Tissue equivalent material or bolus may also be used to achieve sufficient skin surface dose as radiation beams have varying specific depths at which they deposit maximum dose.^10^, ^11^

Previously, cancers located near the eye have been a contraindication for electron treatment.^12^ This is due to deeper depth electron isodose curves in comparison with SXT which presents a challenge when preserving critical structures such as the lens, which has a low maximum dose tolerance. An alternative is the use of internal eye shields made of tungsten or lead which reduce lens dose through direct attenuation of the treatment beam and hence can reduce the risk of developing complications such as cataracts.^13^, ^14^

Currently, there is limited literature on the use of internal eye shields to mitigate this problem. This has led to possible missed opportunities to offer treatment locally to patients at regional radiation departments across Australia that do not have access to SXT or plastic surgery. This report aimed to describe the process for the use of internal eye shields at GenesisCare Fraser Coast Radiation Oncology (GCFCRO) for periorbital skin cancers and the associated clinical outcomes and patient perceptions of the delivery and results of this procedure.

## Methods

Patients over the age of 18 years who had received electron treatment requiring an internal eye shield as a WBHHS patient at GCFCRO between 1 January 2017 and 1 April 2022 were included in this retrospective data audit. Ethics approval was granted by The Prince Charles Hospital Human Research Ethics Committee (Project ID: 78460). This project was conducted in two phases. Phase I was an audit of the departmental technique and short‐term clinical outcomes from the use of internal eye shield for patients receiving electron treatment for skin cancer near the eyes at GCFCRO in partnership with WBHHS. Phase II was a survey offered to patients from Phase I to report the long‐term outcomes, side effects and patient satisfaction related to the treatment.

### Phase I

**Figure 1:**
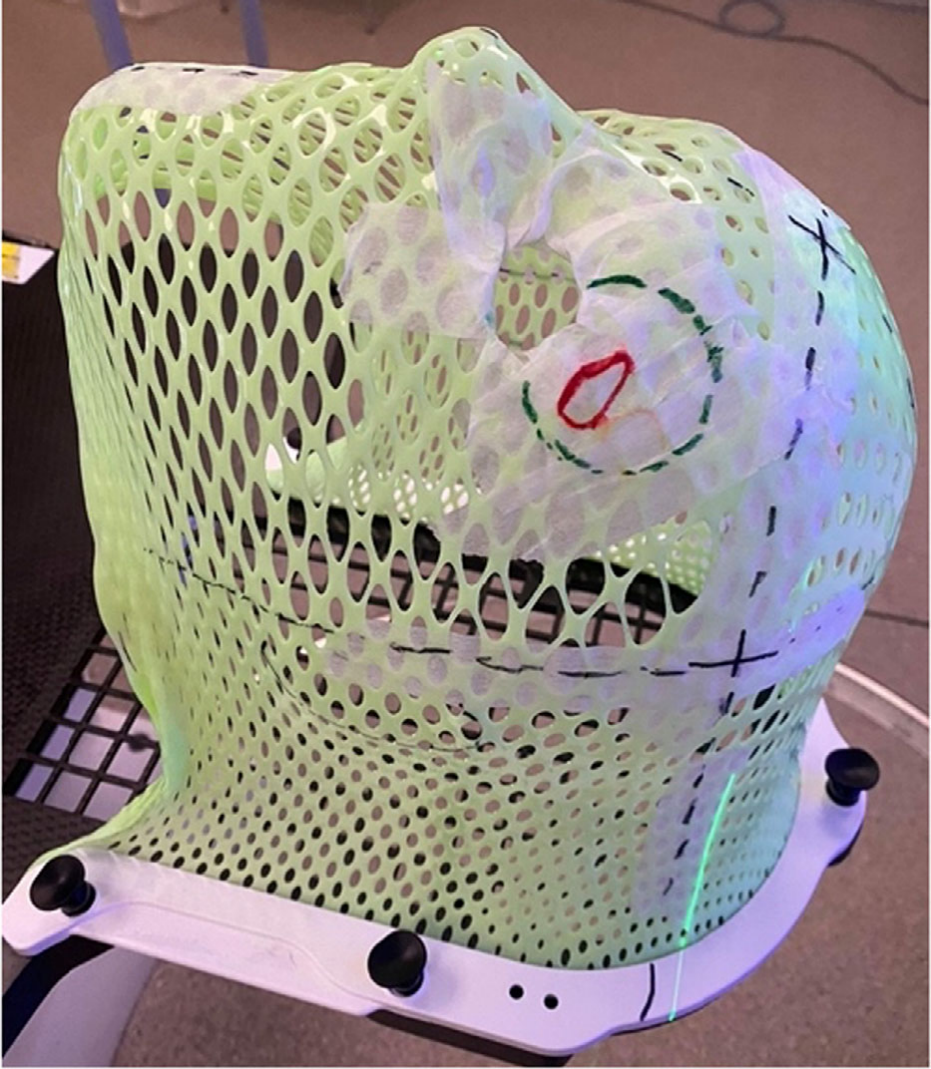
Immobilisation mask with hole cut out for eye shield positioning. Green pen marks represent the treatment field.

**Figure 2:**
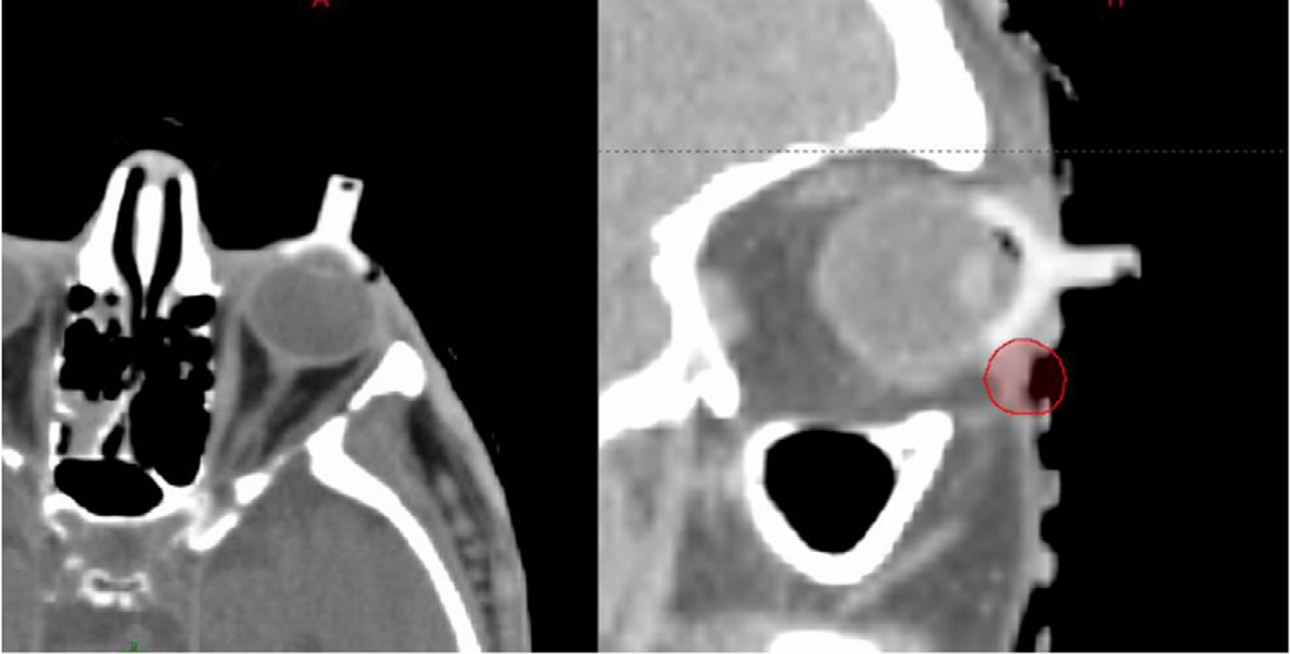
Dummy eye shield inserted for patients CT scan. Red circle is the clinical target volume.

Care for all patients requiring an internal eye shield for their EBRT at GCFCRO was delivered as per current routine protocol. The initial patient consultation was at WBHHS with one of the GenesisCare ROs and was followed by a simulation session. This session determined the patient and eye shield positioning for their subsequent treatment. 15 minutes prior to simulation, Oxybuprocaine eye drops were administered to numb the eye prior to shield insertion. Only trained staff, approved as competent in this technique, inserted the shield. Training assessment consisted of supervised shield insertion with senior radiation therapist and nursing staff who assessed that a sterile field was maintained, shields were inserted with proficient technique to minimise patient discomfort, and correct treatment was delivered. Once in the computed tomography (CT) scanner, the internal eye shield stem was held in position by a small hole cut out of the thermoplastic shell as seen in Figure 1. Shields are made in two sizes (diameters of 2.1 cm or 1.7 cm). After considering the size of the patient's eye, the proximity to the treatment area and patient comfort, the larger shield was preferred if deemed suitable due to the increased protection it offered. A ‘dummy’ internal eye shield, as seen in Figure 2, made of plastic was used for this simulation to avoid the creation of metal artefact on the planning scan which causes difficulty visualising the soft tissue surrounding the shield.

All patients received a planning CT using a Siemens CT scanner (Siemens Healthcare, Erlangen, Germany) with standard scan limits of the superior aspect of the headboard to the inferior portion of the chin, using 1.5 mm slices and a 500 field of view. From this scan the lens, lacrimal gland and target volume were delineated and a patient‐specific plan created. Dose and fractionation were determined by the RO. All plans were calculated in Eclipse 16.1 (Varian Medical Systems, Inc.) planning system.

Once the plan had been calculated, the effect of the eye shield on the lens dose was determined. This was achieved by two techniques: (1) manual calculation using the transmission factor associated with the size and number of shield caps multiplied by the lens dose calculated in Eclipse or (2) the eye shield stem and base overridden to 13,430 and 23,581 HU, respectively, and the Eclipse calculation used. After this, physics reviewed the dosimetry of each treatment plan and determined whether one or two caps made from anodised aluminium were to be placed over the lead eye shield prior to insertion.

All treatments were delivered by a Elekta Versa HD linear accelerator (Elekta AB, Stockholm, Sweden) using a fixed angle electron technique. A variety of prescriptions were administered at the radiation oncologists' discretion depending on the tumour size and site, plus the patient's residential location and compliance. For example, elderly patients requiring transport to travel over 1 hour each way to the department would be suitable for a shorter course of treatment delivered twice or three times a week. The eye shield was placed by one of the trained staff centrally in the respective eyelid and slight positional adjustments made to fit the hole of the mask. After completion of the scan and each treatment, shields were sent to a central sterile supply department for sterilisation daily. This department had two large and two small shields and one of each large and small dummy shields to ensure multiple patients could receive their course at the same time and if there were any delays in sterilisation.

### Phase II

Patients from Phase I, whose EBRT treatment had been completed before the end of 2021 were invited to be involved in Phase II of the study. A hands‐off approach to recruitment was used with patients being contacted by a person external to GCFCRO to ensure they did not feel obliged to participate.

Patients had three options for participation: (1) To agree to participate in the survey including having clinical identifiable photographs taken and to consent for the survey results and photographs to be published, (2) to agree to participate in the survey including having photographs taken and to consent for the survey results only to be published and (3) to agree to participate in the survey only.

All patients who consented were also given the option to withdraw their consent at any time. Patients could either complete the survey via phone or face‐to‐face interview. Patients surveyed varied from 6 months to 5 years post‐treatment.

The survey consisted of 13 questions relating to patient satisfaction and treatment outcomes with a combination of, ranked and multiple‐choice questions with open‐text comments responses.

### Data collection and statistical methods

Data were collected and collated using Microsoft Excel (Microsoft Corporation, WA). The Checklist for Reporting of Survey Studies (CROSS) was used with descriptive statistics of all data including frequency and percentages reported. Specifically:

#### Phase I

Patient health records and information were retrieved via GCFCRO databases ‘Mosaiq (Elekta AB, Stockholm, Sweden)’ and ‘Eclipse 16.1 (Varian Medical Systems, Inc.)’. The data were descriptively analysed into tables displaying age, gender, site of tumour, history of skin cancers, histology, previous surgery, volume of treatment area, dose and fractionation, energy, lens dose before and after transmission factor applied and short‐term side effects experienced.

#### Phase II

Eligible patients were contacted and invited to complete a survey via an online Zoom meeting or a face‐to‐face interview. This survey was developed by the research team, content validity assessed by radiation oncologists and approved for use by the research ethics committee. Patients were asked to rank the functional and clinical outcomes and their satisfaction of the treatment on a scale of 1–5, 1 being not satisfied (poor) outcome, 3 being reasonably satisfied and 5 being very satisfied (excellent) outcome.

A set list of 13 questions were asked to all patients (see Appendix S1) with responses either multiple choice with open‐text comments or ranking on a scale of 1–5. Multiple‐choice questions included: treatment short‐ and long‐term side effects; and if the patient: required sedative medication; thought having this treatment option locally beneficial; would have this treatment again; would recommend this treatment to friend/family and have surgery prior to radiation therapy. Open‐text questions asked who referred the patient to radiation oncology and short and asked for comments regarding comparing surgery with radiation treatment if the patient had had previous surgery for a skin cancer. Open‐text responses were collated and frequency analysis conducted. The remaining ranked questions were: how would you rate treatment efficiency (time spent in the room), invasiveness of the mask, invasiveness of the eye shield, treatment duration (length of entire treatment course), cosmetic outcome, comparison of radiation to surgery if applicable.

The cosmetic outcome was also rated (on a scale of 1–5) on the same scale used by the patients, by an independent researcher (third year medical student with no oncology background). The independent researcher was invited to rate some ‘mock’ cosmetic results first and results discussed. When consensus was reached, and the independent researcher responses were consistent they were asked to rate the cosmetic outcome of the treatment of the patient photographs without knowledge of the patient's own ranking.

## Results

### Phase I

Between January 2017 and April 2022, 17 patients had electron treatment for NMSC requiring an internal eye shield at GCFCRO. 15 patients were referred to radiation oncology by their local general practitioner, one by their RO and one by a surgeon. The median age was 72, (range 49–93) with 70% (*n* = 12) being male (See Table 1). The most common treatment site was the lower eyelid. Nine patients had right eye involvement and eight had left eye involvement with one patient having both eyes affected. There were 10 (59%) BCCs and seven (41%) SCCs. The majority (*n* = 8, 47%) of patients received 40Gray (Gy) in 10 fractions (#), with doses ranging from 30Gy to 50Gy in 5# to 20#. Minimum field size was a 3 cm circle. All plans met lens and lacrimal gland tolerances of maximum ≤8 Gy and mean ≤30 Gy, respectively.

**Table 1:** Demographics and treatment details of patients treated with electron beam ration therapy in a regional centre for skin cancer near the eye (*n* = 17).

	Total (*n* = 17)	Completed survey (*n* = 9)
	Frequency (%)	
Gender		
Male	12 (70)	8 (89)
Female	5 (30)	1 (11)
Age in years (median; (range))	72; (49–93)	66; (50–82)
History of skin cancers		
No	8 (47)	0 (0)
Yes	9 (53)	9 (100)
Histology		
Basal cell carcinoma	10 (59)	4 (44)
Squamous cell carcinoma	7 (41)	5 (56)
Previous surgery		
No	9 (53)	3 (33)
Yes	8 (47)	6 (67)
Treatment location		
Inner Canthus	4 (23)	2 (22)
Outer Canthus	3 (18)	1 (11)
Lower eyelid	8 (47)	5 (56)
Upper eyelid	2 (12)	1 (11)
Prescription of radiation		
50 Gy/20#	2 (12)	1 (11)
45 Gy/15#	4 (23)	2 (22)
40 Gy/10#	8 (47)	4 (45)
42.25 Gy/15#	1 (6)	1 (11)
40.5 Gy/9#	1 (6)	1 (11)
30 Gy/5#	1 (6)	0 (0)
Size of shield		
Large	1 (6)	1 (11)
Small	8 (47)	5 (56)
Not documented	8 (47)	3 (33)
Number of caps		
0	3 (18)	1 (11)
1	8 (47)	5 (56)
2	2 (12)	2 (22)
Not documented	4 (23)	1 (11)

All patients experienced a skin reaction with Grade 1 or Grade 2 erythema reported in 8 (47%) and 9 (53%) of patients, respectively (see Table 2). 59% (*n* = 10) of patients had dry/scratchy eyes towards the end of their treatment. Short‐term side effects collected from patient notes upon their weekly nursing review highlighted that a mild reaction of the sclera which was typically mild and relieved with topical agents was reported in 71% (*n* = 12) of patients. Only 12% of participants were fatigued by the end of their treatment and all patients who received treatment to the inner canthus had some temporary nasal blockage. The management of these acute side effects were QV lotion and Flamigel for skin reactions, Chlorsig eye drops for dry eye and Oxybuprocaine for pain relief. No patients required sedation at any point of simulation or treatment.

**Table 2:** Short‐ and long‐term side effects of patients treated with electron beam ration therapy in a regional centre for skin cancer near the eye (*n* = 17).

	Total (*n* = 17)	Completed survey (*n* = 9)
	Frequency (%)	
*Side effects: Early toxicity*		
Skin erythema (highest grade)^1^		
Grade 1	8 (47)	No: 5 (55)
Grade 2	9 (53)	Yes: 4 (45)
Dry/scratchy eyes		
No	10 (59)	5 (55)
Yes	7 (41)	4 (45)
Sclera reaction		
No	5 (29)	
Mild and relieved by topical agents	12 (71)	
Fatigue		
No	15 (88)	6 (67)
Yes	2 (12)	3 (33)
Nasal blockage		
No	15 (88)	8 (89)
Yes	2 (12)	1 (11)
*Side effects: Long‐term/late toxicity*		
Blurred vision		
No		8 (89)
Yes		1 (11)
Photosensitivity		
No		7 (78)
Yes		2 (22)
Watery/itchy eye		
No		7 (78)
Yes		2 (22)

^1^ Severity not asked in Phase II.

### Phase II

Of the 15 patients from Phase I who finished their treatment before 2021, six patients consented to interview in person with consent given for clinical photographs to be published, three consented to a phone interview and the remaining patients were unable to be contacted (*n* = 4) or passed away of other causes (*n* = 2). All patients who were able to be contacted, agreed to answer the survey.

Results from Phase II detailed high patient satisfaction with functional and cosmetic outcomes. All patients agreed (100% responded yes compared to no) that it was beneficial to have this treatment option locally, would have it again and would recommend it to family and friends. Three of the patients surveyed had undergone surgery for the cancer prior to treatment and all agreed they preferred EBRT over surgery.

**Figure 3:**
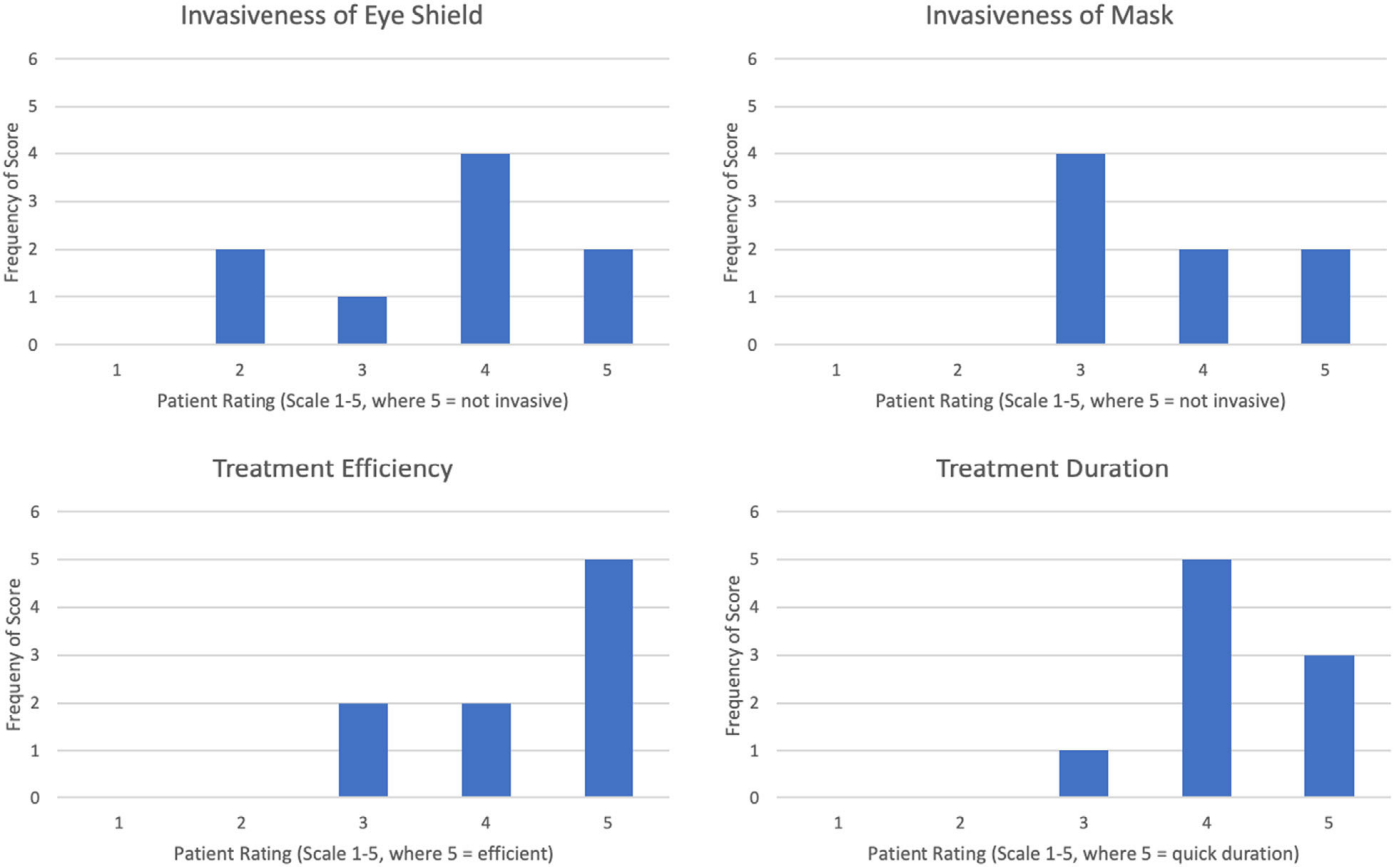
Patient rankings for invasiveness of mask and eye shield and treatment efficiency and duration, where 1 is unsatisfactory (poor/invasive) outcome, 3 is a satisfactory outcome and 5 excellent (very satisfied, non‐invasive) outcome.

One patient did not have a mask for treatment, but overall patients rated the invasiveness of the eye shield lower than the invasiveness of the mask, with 7 (78%) of patients satisfied with the invasiveness of the eye shield compared to all 8 (100%) not finding the mask invasive (Figure 3). Treatment efficiency and duration were ranked as satisfactory or higher by all participants.

Patients were asked to recall any short‐term side effects and management they received and only two of the nine participants were unable to. Patient side effect responses corroborated with the short‐term side effects recorded in the clinical audit from Phase I. All participants were also asked to list their long‐term side effects. Two of the nine participants experienced ongoing long‐term side effects of blurred vision, photosensitivity and watery/itchy eye. These were managed with eye drops and ongoing review with their RO. One patient had eyebrow alopecia. The remaining patients did not experience any long‐term side effects.

Cosmetic outcomes of the six patients who consented to photographs were rated by an external reviewer. These outlined high results with five out of six being rated as excellent.

**Figure 4:**
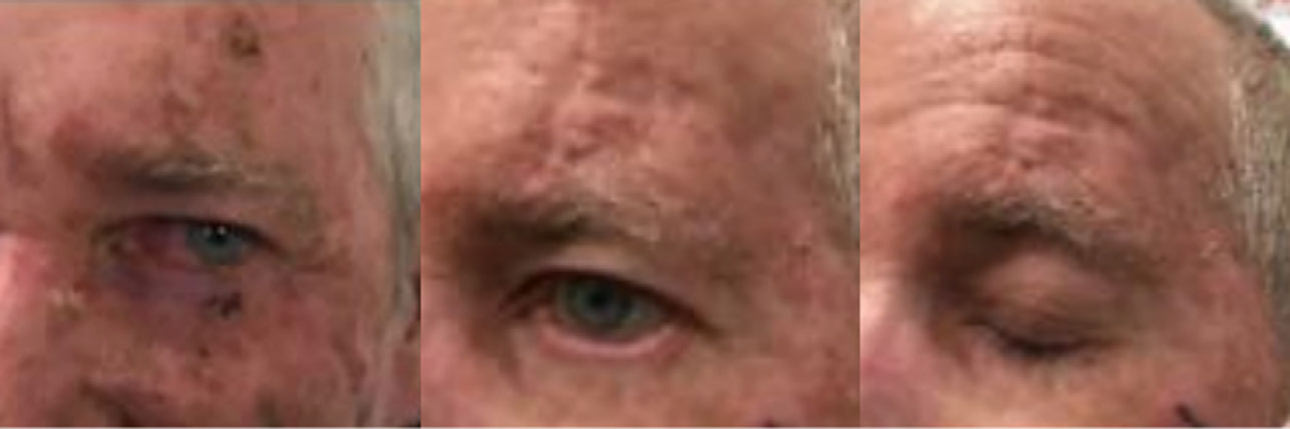
Before and after EBRT clinical photographs of left lower lid cancer. Permission obtained from patient to publish photograph.

Pre‐ and post‐RT photographs can be seen below for two patients with similar ongoing ectropion of the treated eye. The patient shown in Figure 4 also has ongoing photosensitivity and rated their outcome poorly (score of 2) with the external reviewer rating it as a 5.

**Figure 5:**
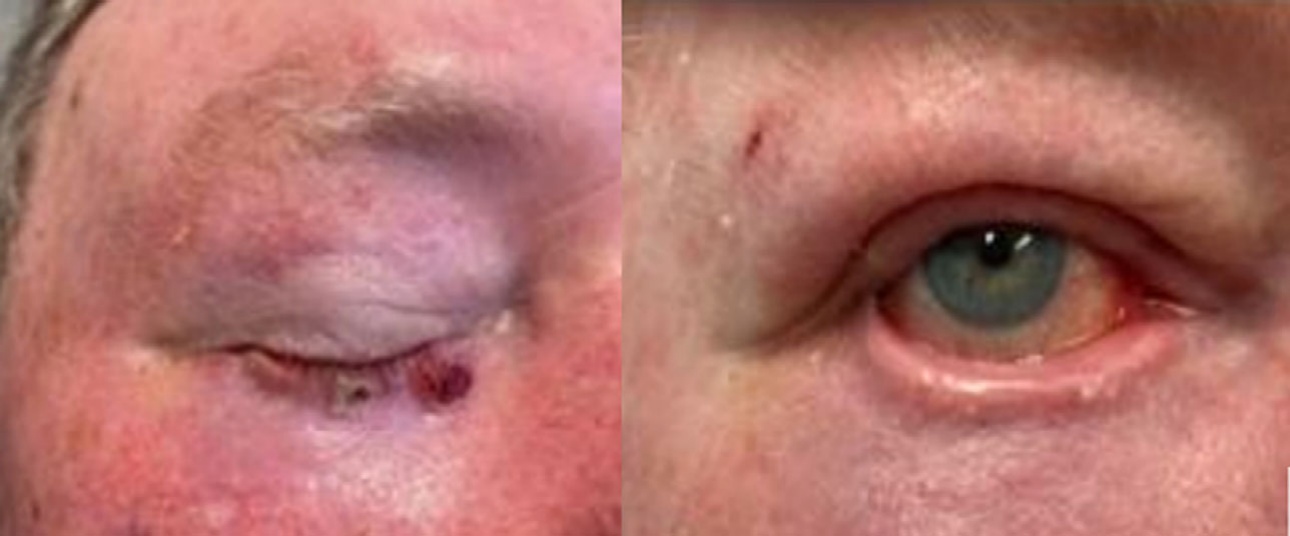
Before and after EBRT clinical photographs of right inner canthus cancer. Permission obtained from patient to publish photograph.

The patient shown in Figure 5 with a similar tumour and not experiencing any visual disturbances, rated his outcome a 5 which was comparable to the external reviewer rating of 4.

## Discussion

This study has produced some key information describing the process and potential benefits of the use EBRT and internal eye shields in a regional centre. There is limited research regarding the use of internal eye shields for electron beam EBRT as the preferred radiation modality for skin cancer near the eye. As outlined by Webster and Johnson^3^ and Mirimanoff and Sozzi^12^ the preference for using SXT is due to the shallow depth and small penumbral characteristics of orthovoltage treatment, which allows increased dose sparing to sensitive normal tissues located around the eye. However, many regional centres including the one involved in this study do not have access to SXT or plastic surgery. Thus, reporting on the methods and patient outcomes when using internal eye shields for electron beam EBRT is important to ensure high‐quality equitable patient outcomes are achievable regardless of a patient's place of residence.

We have shown that electron beam EBRT requiring an internal eye shield highlighted a consistency in technique of our study to that described in the literature. Internal eye shields made from lead or tungsten are effective in avoiding acute and late side effects to the underlying organs.^4^, ^10^ Shiu et al.^15^ suggest the use of tungsten shields as being superior over lead shields to ensure adequate protection of ocular structures. This work also highlighted the benefit of using large shields over small to increase organ protection, consistent with our methods.

The use of eye shields in the planning process is also discussed in research to ensure an accurate representation of the shield, underlying organs and skin cancer for the planning process. Metallic structures made of high atomic number materials greatly attenuate a kV photon beam from the CT, resulting in artefact around the metallic structures.^13^ A high‐quality scan is required to ensure accurate outlining of the critical structures and patient dose calculation and although the scans can be post‐processed to help remove this artefact, a dummy shield made from plastic removes any artefact in the scan. This method was suggested by Park et al.^13^ and Kang et al.^16^ so accurate patient dose calculation can be achieved in the treatment planning system.

Investigating patient satisfaction relating to this treatment technique and their perception of their clinical outcomes, was an important and unique part of this study. As Leech et al.^17^ highlights, it is important to study patient opinion and decision making, as by including patients as active participants in their health care, not only does this increase satisfaction, but also it increases adherence to treatment, overall outcomes of care and can reduce decision regret. 89% of all participants rated their overall cosmesis as satisfactory or higher and all were rated as highly satisfactory/excellent by an external impartial reviewer. During the survey all patients who had undergone surgery to their lesion prior to EBRT, detailed their preference of having EBRT alone. A previous study by Frank and Mahajan^4^ showed no significant difference in local, regional and overall disease‐free survival rates at 5 years between patients treated with RT alone versus those having surgery. Thus, the results of this investigation in conjunction with research by Webster and Johnson^3^ demonstrate the effectiveness and suitability of EBRT over surgery for treatment of skin lesions near the eye.

It is, however, important to note that patients with ongoing long‐term side effects rated their experience with an overall lower cosmesis score. All patients surveyed who have continued to have dry eye or visual disturbances particularly sensitivity to light rated their overall cosmesis reasonably satisfied. The external rating of these two patients based only on post‐treatment photographs was very satisfied.

An important aspect identified from the patient survey responses was the benefit of being able to receive cancer treatment locally, with 100% of patients surveyed responding yes compared with no when asked if having this treatment option locally was beneficial to them. Care close to home increases patient satisfaction, adherence and treatment outcome and thus, increasing access to healthcare services for regional and rural Australians is an increasingly important goal within and beyond the radiation oncology setting.^18^, ^19^ Thus this underscores the importance of having this technique as an available treatment option at regional radiotherapy departments to improve access to care and patient‐related outcomes which are not directly attributed to the intervention itself but rather the processes surrounding that care such as; travel, follow‐up appointments, associated costs and psychological burden of needing to travel for treatment.

A future direction for this study would be to reconsider the question asked to patients to include further details such as weighing up risks/benefits, cosmetic outcomes and logistics and cost of travel and using a Likert scale response instead of yes or no questions to decrease result bias and increase data quality. Whilst the survey in this study was deliberately kept simple to encourage patient participation, it would additionally be interesting to dissect the topic of care closer to home in an interview setting to allow thematic responses to be identified from qualitative data.

The researchers would like to acknowledge that there are several limitations to this study. Firstly, a large variety of dose and fractionations were reported. These were all determined by the treating oncologist and was dependent on tumour site and size as well as patients age and place of residence. A larger dose per fraction treated two to three times a week was used for elderly patients travelling large distances to minimise the impact on their life. This was also outlined in Webster and Johnson^3^ research detailing, although the standard 2Gy/fraction resulted in better cosmetic results and less necrosis, it must be weighed up with the considerable logistical problems of mobilising elderly patients for treatment.

A limitation of the research was the proportion of patients unable to be contacted which prevents generalisation of the results but despite this being a small sample size, the team believes the positive patient satisfaction levels collected show the benefits of this technique and facilitates other regional centres to offer this care to their patients.

A further limitation was that all patients surveyed had finished their course of treatment at least 6 months previously, time ranging from 6 months to 4 years. Having such a varied time post‐treatment for patients being interviewed could have led to recall bias with those having completed their radiation for a longer period of time unable to specifies long‐term side effects and management as accurately. Interestingly, patient reports of short‐term side effects and management did not match what was recorded in their clinical notes from the audit conducted in Phase I in only two of the nine patients, suggesting the time between treatment and survey had a limited impact on their reporting bias.

## Conclusion

This study summaries the protocol used for internal eye shield treatments at GCFCRO and patients' perceptions of clinical outcomes and satisfaction of this service. Results indicate that EBRT with the use of internal eye shields is an acceptable alternative modality for the treatment of SCCs and BCCs around the eye. Satisfactory dosimetry was achieved with this technique with limited acute and chronic side effects reported by patients. Importantly, results from Phase II of this investigation suggest patient preference to receive care close to home. In Australia, regionally based patients do not have easy access to plastic surgery or SXT and prefer receiving treatment locally due to the cost and inconvenience of travelling to major metro centres for such treatment. Reporting on the both the treatment protocol used for internal eye shield treatments at GCFCRO, and patient satisfaction from this technique may inform other centres interested in adopting this treatment protocol where surgical and superficial modalities are not available or appropriate.

## Conflict of Interest

Authors declare that there is no conflict of interest.

## Supporting information


**Appendix S1.** Survey questionnaire.

## Data Availability

The data that support the findings of this study are available on request from the corresponding author. The data is not publicly available due to privacy or ethical restrictions.
