# Supplementary material for: Evaluation of the clinical outcomes and patient satisfaction related to the use of internal eye shields for electron external beam radiation therapy
Source: J Med Radiat Sci. 2024 Jul 15;71(4):555–63. doi: 10.1002/jmrs.812 (PMC11638373; doi:10.1002/jmrs.812)
Supplement: Supplementary file 1 — Appendix S1. Survey questionnaire. [file JMRS-71-555-s001.pdf]

SURVEY QUESTIONNAIRE

Clinical Review of Internal Eye Shields for External Beam Radiation Therapy

Thank you for your participation. This questionnaire takes about 5 minutes to complete.

Considering your radiation treatment to your eye area:

1) On a scale of 1-5, 1 being not satisfied (poor) outcome, 3 being good (reasonably satisfied) and 5 being an excellent (very satisfied) outcome, how would you rate the;

   a. Treatment efficiency (time spent in the room each day)
   Not satisfied ☐ 1 ☐ 2 ☐ 3 ☐ 4 ☐ 5 Very satisfied

   b. Invasiveness of the **mask**
   Very invasive ☐ 1 ☐ 2 ☐ 3 ☐ 4 ☐ 5 Not invasive

   c. Invasiveness of the **eye shield**
   Very invasive ☐ 1 ☐ 2 ☐ 3 ☐ 4 ☐ 5 Not invasive

   d. Treatment duration (duration of entire treatment course)
   Not satisfied ☐ 1 ☐ 2 ☐ 3 ☐ 4 ☐ 5 Very satisfied

2) How would you rate your cosmetic outcome of treatment?
   Not satisfied ☐ 1 ☐ 2 ☐ 3 ☐ 4 ☐ 5 Very satisfied

3) Did you require sedative medication during your treatment?
   ☐ yes
   ☐ no

4) Do you feel having this treatment option locally was beneficial to you compared to surgery or travelling to a larger centre?
   ☐ yes
   ☐ no

5) What short term side effects did you have during and shortly after the treatment?
   ☐ fatigue
   ☐ skin reaction/redness of skin
   ☐ dry eye
   ☐ weeping eye
   ☐ pain
   ☐ Nasal blockage
   ☐ other (please specify)

   ____

6) How did you manage your short-term side effects?
- ☐ Chlorsig eye drops
- ☐ pain relief
- ☐ QV moisturiser
- ☐ Flamigel
- ☐ Dressing
- ☐ Nasal spray
- ☐ other (please specify)

____

7) What long term side effects did you have?
- ☐ dry eye
- ☐ vision disturbance
- ☐ hair loss
- ☐ cataracts
- ☐ other (please specify)

____

8) How did you manage your long-term side effects?
- ☐ Eye drop
- ☐ other (please specify)

____

9) Would you have this treatment again for a similar cancer?
- ☐ yes
- ☐ no

10) Who referred you to radiation oncology?

Please specify: ______________________________________

11) Would you recommend this treatment to friend/family if they had a skin cancer in a similar area?
- ☐ yes
- ☐ no

12) Did you have surgery before your radiation therapy for your skin cancer?
- ☐ yes
- ☐ no

13) If you have had previous surgery, how did this treatment compare? Where 5 is would prefer to have radiotherapy over surgery.

Would prefer surgery ☐ 1 ☐ 2 ☐ 3 ☐ 4 ☐ 5 Would prefer radiotherapy.

Comment: ______________________________________
